# [Retracted] Glutathione peroxidase 2 overexpression promotes malignant progression and cisplatin resistance of KRAS‑mutated lung cancer cells

**DOI:** 10.3892/or.2024.8834

**Published:** 2024-10-29

**Authors:** Mei Wang, Xu Chen, Guang Fu, Mingjian Ge

Oncol Rep 48: 207, 2022; DOI: 10.3892/or.2022.8422

Following the publication of this paper, it was drawn to the Editor's attention by a concerned reader that certain of the BrdU incorporation assay data shown in Fig. 7C were strikingly similar to data that had already appeared in another article written by different authors at different research institutes [Lu M, Qin X, Zhou Y, Li G, Liu Z, Geng X and Yue H: Long non-coding RNA LINC00665 promotes gemcitabine resistance of Cholangiocarcinoma cells via regulating EMT and stemness properties through miR-424-5p/BCL9L axis. Cell Death Dis 12: 72, 2021].

Owing to the fact that the contentious data in the above article had already been published prior to its submission to *Oncology Reports*, the Editor has decided that this paper should be retracted from the Journal. The authors were asked for an explanation to account for these concerns, but the Editorial Office did not receive a reply. The Editor apologizes to the readership for any inconvenience caused.

